# In vitro assessment of fracture resistance of endodontically treated teeth restored with short fiber reinforced resin based composite and ceramic overlays

**DOI:** 10.1186/s12903-025-05480-x

**Published:** 2025-02-08

**Authors:** Mona Elshirbini Hafez, Ahmed Ata Abd El-ghany, Ahmed Ismail Taha, Ashraf Amin

**Affiliations:** 1 Faculty of Dentistry, Kafr Elsheikh University, Kafr Elsheikh, Egypt; 2 Faculty of Dentistry, Al-Azhar University, Cairo , Egypt

**Keywords:** Fracture resistance, Fiber reinforced resin, Onlays, Endodontically treated teeth

## Abstract

**Background:**

Conventional full-coverage crowns are used after endodontic treatment to enhance the fracture toughness of these teeth. However, these crowns lead to extensive loss of tooth structure. Continuous trials to develop a biomimetic conservative direct restorative material that can be used as a dentine substitute led to the development of the short fiber reinforced resin composite.

**Methods:**

Fifty extracted intact upper premolar teeth were selected. Ten teeth serve as positive control without any treatment (Group 1). The remaining forty teeth were received root canal therapy. Standardized MOD cavities were prepared in 30 teeth of them, then they were randomly divided into three groups: Group 2: teeth received no coronal restorations; Group 3: MOD cavities restored with packable short fiber reinforced composite (EverX Posterior), followed by 2 mm nanocomposite; and Group 4: MOD cavities restored with flowable short fiber reinforced composite (EverX Flow), followed by 2 mm nanocomposite. For the remaining ten teeth, MOD cavities were prepared, and cusps were reduced to receive ceramic overlays (CeraSmart 270). After thermocycling, fracture load is applied using the Instron Universal machine until samples fracture. Fracture loads were recorded, and fracture mode was evaluated. All data were subjected to statistical analysis.

**Results:**

Teeth restored with CeraSmart 270 overlays and those restored with high aspect ratio short fiber-reinforced resin composite recorded the highest resistance to fracture with no significant differences between them.

**Conclusions:**

EverX Posterior may be considered a conservative substitute for indirect overlays to restore endodontically treated teeth with MOD cavities.

**Clinical relevance:**

Clinically, using fiber reinforced composite as a substitute for indirect restorations preserves the tooth structure and provides proper resistance to fracture.

## Background

Endodontically treated teeth have less fracture resistance than vital teeth; this is primarily attributed to the loss of key anatomical crown structures. Caries, access cavities, and root canal preparations are the primary factors contributing to tooth fragility and structure loss [1, 2]. Extensive cavity preparations such as Mesio-Occluso-Distal Cavities (MODs) remarkably decrease tooth strength, which may reach a 54% reduction compared to unprepared teeth. Consequently, MOD cavities may lead to detrimental occlusal force effects as micro-fractures [2].

Conventional full-coverage crowns are used for coronal coverage after endodontic treatment to enhance fracture toughness and ensure the durability of restorations. However, the extensive tooth reduction required for these crowns leads to significant loss of tooth enamel and dentin [3]. Adhering to recently advanced minimally invasive concepts is considered the optimal method to restore and preserve the structure of endodontically treated teeth [4].

In an attempt to preserve the tooth structure and substitute indirect restorations while decreasing polymerization shrinkage and boosting the fracture toughness of resin-based composites, an innovative fiber-reinforced composite is developed by adding fibers to the main matrix. Various types of fibers, such as glass, carbon, or polyethylene fiber, can be incorporated into these composites [5].

According to biomimetic dentistry, there is no need for rigid materials to withstand masticatory forces. The primary objective is to replace lost hard tooth tissues, such as dentin and enamel, with restorative materials that closely resemble natural tissues in terms of their mechanical characteristics [6]. Pascal Magne’s early research suggests that highly filled laboratory composites or feldspathic porcelain are the best materials to replace brittle, rigid enamel. In contrast, microhybrid composite resin should be used to replace dentin [7]. In 2000, multiple research studies highlighted the significance of a third kind of tissue, or layer, known as the dentin-enamel junction (DEJ) [7, 8]. The high toughness and tensile strength of dentin have been attributed to the high aspect ratio collagen fibers.

In response to the ongoing research to develop a dentin substitute, EverX Posterior GC, a biomimetic restorative material, was introduced in 2013. The in-continuous high aspect ratio, millimeter-scale fibers included within this material combine E- glass with barium glass fibers, which are randomly oriented inside the resin, creating this fibrous composite. By using this orientation, the manufacturer claims to get isotropic reinforcement in multiple directions and potentially match the toughness of natural dentine. The purpose of this composite was to replace the lost dentin with a material that behaves similarly. Clinical research has also demonstrated that this material can concurrently imitate the stress-absorbing qualities of the DEJ. Its fibrous characteristic mimics natural dentin in its collagenous fibrous structure, which prevents the cracks beginning in the enamel from propagating into the dentin [9, 10]. With a flexural strength of 155 MPa and a fracture toughness of 4.7 MPa, the mechanical properties of the short fiber-reinforced composite continue to ensure its role as the most biomimetic restorative material [11–13].

Some studies showed that short fiber-reinforced resin-based composites are capable of stopping the propagation of cracks and boosting the fracture toughness of restoration. This material can be cured in bulk up to a 4-mm thick increment [14–16]. Faced with the compromised aesthetics and workability of EverX Posterior, the manufacturer launched a new low-viscosity version in 2019.

The new flowable version, named EverX Flow, is designed to address the shortcomings of its predecessor and features a dentin color and enhanced handling. This is achieved by reducing the size of the e-glass fibers to the micrometer- scale and increasing the content of fibers up to 25% by weight. The manufacturer claims to get better fracture toughness than the packable old version [17].

CAD/CAM materials and techniques were proven to provide proper control and benign distribution of the stresses facing extensive MOD restorations, as they offer excellent machinability, wear resistance, aesthetics, time savings, and less abrasiveness to opposing teeth [18, 19]. CeraSmart 270, a newly introduced CAD/CAM-based hybrid nano-ceramic, was selected for this study due to its demonstrated superior resistance compared to fracture among other indirect materials tested in previous studies [11, 20].

Nowadays, a difficult economic crisis and a high cost of living are being faced. Patients are seeking affordable and suitable restorations. Substituting indirect ceramic restorations with short fiber-reinforced composite in extensive restorations may act as a dentin substitute and fulfil this popular demand. In this respect, and with the launch of new flowable short fiber-reinforced restorations, there is a scientific gap and an absence of proof related to the material’s suitability as a coronal restoration for endodontically treated premolar teeth. To our knowledge, there is no study comparing the fracture resistance of endodontically treated upper premolars restored with EverX Flow with that restored with indirect restorations. The current study’s rationale is to assess and compare the fracture resistance and failure mode of endodontically treated maxillary premolars restored with two types of direct short fiber-reinforced composite and indirect ceramic overlay restorations. The null hypothesis tested is that there is no difference in fracture resistance or fracture mode between endodontically treated premolars restored with short fiber-reinforced composite, either flowable or packable, or indirect ceramic overlays.

## Materials and methods

### Sample size calculation

Based on the KS test that was performed to examine the normality of the data provided from a prior study by Eaban (2017), the results revealed normally distributed data. The level of confidence was 95%. The power was 80%. According to the significance level, the sample size of this study was 10 per group [21].

**Table 1:** Presented all used materials, their compositions, and manufacturers

Material	Composition	Manufacturer
EverX Posterior	**26wt% resin matrix composed of PMMA**,** bis-GMA and TEGDMA** **Fillers: barium borosilicate 68wt**,** 0.7 μm in size** **E-glass milimeter-scale short fibers total 9wt% with average size 1.3 mm**	**GC Co**,** Tokyo**,** Japan**
EverX Flow	**26wt% resin matrix composed of UDMA**,** bis-GMA and TEGDMA.** **Fillers: barium borosilicate 68wt**,** 0.7 μm in size** **E-glass micrometer-scale short fibers total 25wt% with average size 0.2–0.3 mm**	**GC Co**
Cerasmart 270 (resin matrix ceramic block)	**UDMA**,** Bis-MEPP**,** DMA**,** 71 wt% silica**,** barium glass**	**GC Co**

Abbreviations: PMMA: poly methyl methacrylate; Bis-GMA: bisphenol A diglycidyl methacrylate; TEGDMA: triethylene glycol dimethacrylate, UDMA: urethane dimethacrylate

### Teeth selection

Fifty intact, freshly extracted human single-rooted maxillary upper premolars of comparable size were selected for this study. Extraction of the teeth was performed in the Department of Oral Surgery, Faculty of Dentistry, Kafr Elsheikh University, for either orthodontic or periodontal purposes for patients between 35 and 45 years of age. The dimensions of crowns of teeth included in the study were 9.0–9.4 mm buccolingually, 6.8–7.1 mm mesio-distally, and 7.9–8.5 mm cervico-occlusally. All teeth were inspected under magnifying loups of 2.5X (Smedent, China) to ascertain and rule out teeth with faults or fractures. Using an ultrasonic scaler with water coolant, calculus and retained tissues were removed from the teeth, and then all teeth were stored in a saline solution until the experiment.

### Specimens grouping

Using Random.org software, the selected teeth were randomly divided into five groups (10 teeth in each group):

#### Group 1

no endodontic treatment or cavity preparation was performed (positive control group).

#### Group 2

premolars were endodontically treated; MOD cavities were prepared with no coronal restoration (negative control group).

#### Group 3

premolars were endodontically treated; MOD cavities were prepared and restored with packable short fiber reinforced composite (EverX Posterior), followed by 2 mm nanocomposite.

#### Group 4

premolars were endodontically treated; MOD cavities were prepared and restored with flowable short fiber reinforced composite (EverX Flow), followed by 2 mm nanocomposite.

#### Group 5

**Fig. 1:**
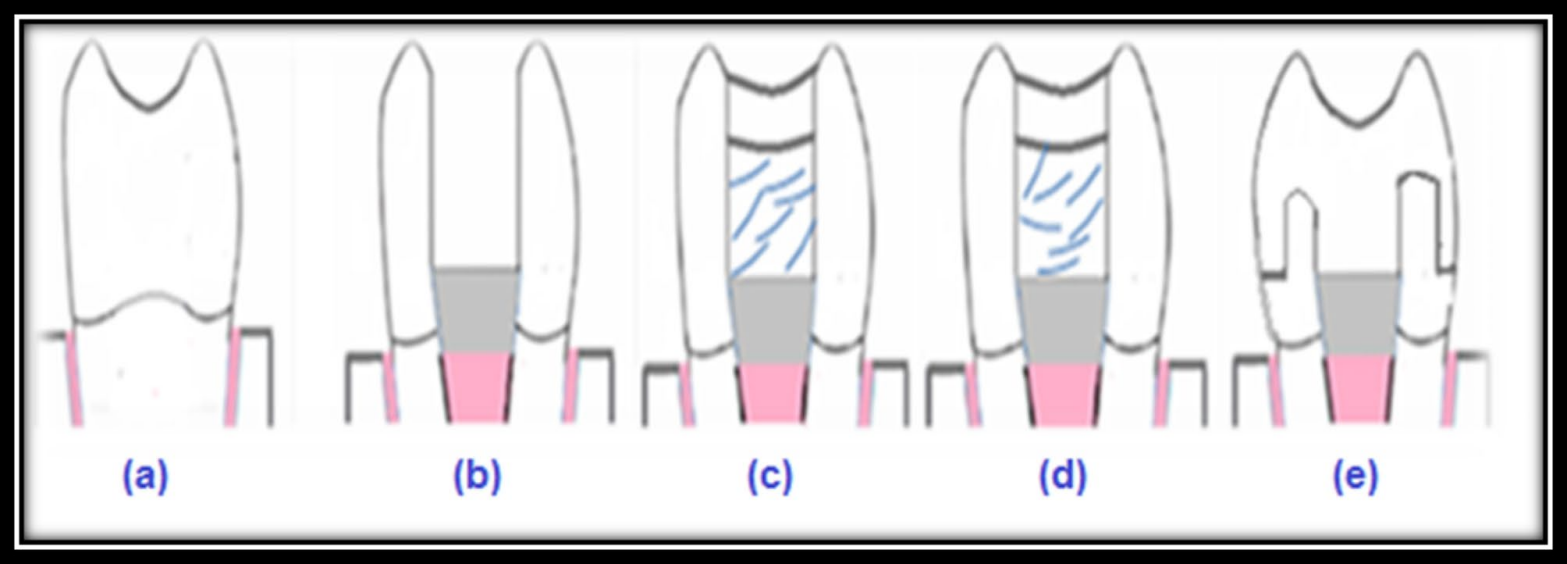
The studied groups (from left to right) **(a)** Group 1: intact teeth; **(b)** Group 2: endodontic treatment and MOD cavity without restoration; **(c)** Group 3: Endodontic treatment, MOD cavity, EverX posterior, nano-composite; **(d)** Group 4: Endodontic treatment, MOD cavity, EverX flow, nano-composite; **(e)** Group 5: Endodontic treatment, MOD cavity, cusps reduction, and CeraSmart overlay

premolars were endodontically treated; MOD cavities were prepared and cusps were reduced. Teeth were restored with a resin matrix ceramic overlay (CeraSmart 270) Figure 1.

### Specimens preparation

Using a thin marker, each selected tooth was demarcated below the cement-enamel junction by 1 mm. The teeth roots were immersed in melted blue wax to the level of the demarcation line. This will provide a wax coating about 0.2 to 0.3 mm thick, covering the root. This thickness nearly mimics the periodontal ligament thickness. The acrylic resin material was mixed until it reached the dough stage and then placed in a polyvinyl chloride tube of 3 cm length and 1.27 cm width. After filling the tube with the resin, the roots were embedded gently in the middle of the acrylic resin cylinder, with parallel angulation to the cylinder’s long axis, until the acrylic resin just covered the line of demarcation. This is nearly the average alveolar bone attachment level to the tooth. In order to standardize the accurate placement and alignment of the teeth inside the cylinder, a custom-made jig was used. After the teeth had been taken from the polymerized resin cylinder, the wax layer was fully melted by dipping the roots in a pot of hot water. To substitute the melted wax layer, an amount of polyether impression material was placed into the empty space created inside the resin after tooth removal. And then the teeth were returned to their place with slight pressure. Any protruding excess polyether material was removed and cleaned [13].

### Endodontic treatment

By one operator, Endo Access Diamond Bur (BR-41, Mani, Japan), in a high-speed handpiece (NSK Nakanishi Inc., Japan), was done to standardize conservative endodontic access cavity preparations in all teeth in groups 2–5. Then start-X tips (Dentsply, Germany) were used to refine the cavities.

To establish the working lengths and confirm the canal patency, #10 K-file (Dentsply) was used. Then sequential enlargement of the root canals was done using Pro-taper rotary instruments from SX to F3, driven by X smart (Dentsply). Canal irrigation with 3 mL of a 2.5% NaOCl irrigation solution was performed after every instrument. When canal preparations were completed, paper points were used to dry the canals. Cold gutta percha cones (Dentsply) impregnated with AH plus endodontic sealer (Dentsply) were laterally compacted to obturate the canals completely. Following the removal of extra gutta-percha with a hot excavator, cotton was used to clean the pulp chamber. Teeth were radiographed to evaluate the accuracy of obturation. A layer of resin-modified glass-ionomer cement (GC Fuji II LC Capsule, GC) was applied to seal the endocavity.

### Cavity preparation

#### For groups 2–4

**Fig. 2:**
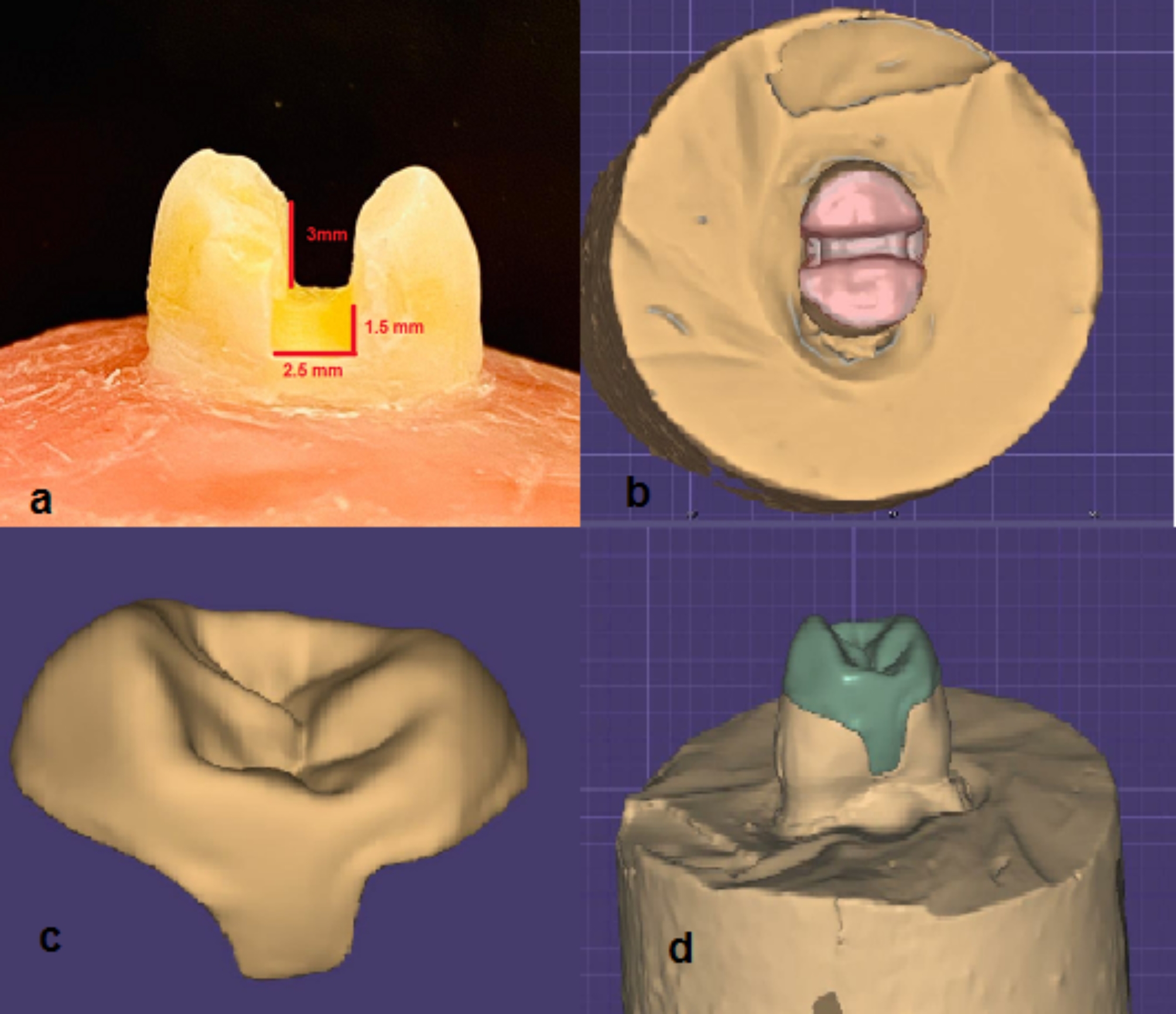
**(a)** MOD preparation design, **(b)** The virtual scan of onlay preparation **(c)** The virtual design of onlay **(d)** Virtual design of onlay adapted to preparation

By one operator, standardized MOD cavities were prepared in all teeth using 1 mm-diameter straight fissure diamond burs (Dentsply Sirona Endodontics, Tulsa, OK, USA) under water coolant. For the occlusal box, the buccopalatal width and depth were 2.5 mm and 3 mm, respectively. The prepared cavity was centralized in the middle third of the tooth, providing a minimum thickness of 2 mm for the remaining buccal and lingual wall thicknesses. The width and depth of the gingival seat were 2.5 mm and 1.5 mm, respectively. 1.5 mm was the length of the axial wall, so the gingival margin was almost a millimeter above the CEJ, keeping the entire cavo-surface margin at 900 (Fig. 2a).

#### For group 5

To evaluate the amount of cusp reduction, templates are constructed by taking silicon impressions of all the teeth in this group before their preparation. Using 80 tapered diamond burs (S 856-016-8 ML, Swiss Tec, Coltene AG, Alstatten, Switzerland), MOD cavities with divergent walls were prepared. Palatal and buccal cusp reductions were 2 mm and 1.5 mm, respectively, following the anatomic contour of the cusps. This reduction ended with a 1.5-thick chamfer placed 2 mm from the tip of the reduced cusps in the palatal and buccal surfaces of the teeth. Using the silicon index and a calliper, the reduction thickness was checked and measured to assure standardization of the reduction. Smoothing of all surfaces and rounding of all internal line angles were performed using a fine diamond bur (Mani dia bur TR-26 F, Mani Inc., Tochigi, Japan). The used burs were changed after each of the five preparations [22].

### Coronal restoration

#### Group 2

No coronal restoration.

#### Group 3

Acid etchant (Etching gel, DMP, USA) was used to etch all the cavity surfaces and left for 15 s, followed by rinsing for an extra 15 s. The dryness step took place gently using an oil-free air blast. Universal adhesive (Scotchbond Universal, 3 M) was applied to all the walls and floors using a micro-brush with vigorous rubbing for 20 s, then light-cured for 20 s. The cavity was filled incrementally and packed with a packable short fiber-reinforced resin-based composite (Everx Posterior, GC, Europe), then cured for 20 s, leaving 2 mm occlusally to be restored with a nano-hybrid composite (Tetric N Ceram ivoclar Vivadent Asia). Finishing and polishing were done using a finishing bur (Dentsply), followed by AL2O3 polishing discs (Soflex TM, 3 M ESPE, St. Paul, MN, USA) in a sequenced order from medium to super-fine.

#### Group 4

Acid etchant (Etching gel, DMP, USA) was used to etch all the cavity surfaces and left for 15 s, followed by rinsing for an extra 15 s. The dryness step took place gently using an oil-free air blast. Universal adhesive (Scotchbond Universal, 3 M) was applied to all the walls and floors using a micro-brush with vigorous rubbing for 20 s, then light-cured for 20 s. The cavity was filled incrementally and adequately packed with flowable short fiber-reinforced resin-based composite (Everx Flow, GC, Europe), then cured for 20 s, leaving 2 mm occlusally to be restored with a nano-hybrid composite (Tetric N Ceram ivoclar Vivadent Asia). Finishing and polishing were done using a finishing bur (Dentsply), followed by AL2O3 polishing discs (Soflex TM) in a sequenced order from medium to super-fine.

#### Group 5

Nano-ceramic CeraSmart 270 blocks were used to construct overlays by machining them using the CAD/CAM machine (CEREC MC XL SW 4.0). The overlays were designed using the Exocad software to standardize the outline of all overlays (Fig. 2. b, c, d). The milling process was performed following the manufacturer’s instructions. Finishing was done using medium and fine silicone points in a low-speed handpiece, followed by final polishing using diamond polishing paste (DIAPOLISHER, GC America, Tokyo, Japan). For the bonding process, 5% hydrofluoric acid for 60 s was used to treat the internal surface of the restorations, followed by rinsing for 60 s and drying for 30 s. The intaglio surface of the overlays was then silanated with Ceramic Primer II and let dry for 60 s [23].

Acid etchant (Etching gel, DMP, USA) was used to etch all the cavity surfaces for 15 s, followed by rinsing for an extra 15 s. The dryness step took place gently using an oil-free air blast for 10 s. Bonding agent was applied, agitated, and polymerized for 20 s, and then all restorations were cemented under pressure.

### Thermo-cycling

Finally, all specimens were stored in deionized water at 37 °C for three days before undergoing thermocycling between 5 °C and 55 °C for 2000 cycles [24].

### Fracture resistance testing

**Fig. 3:**
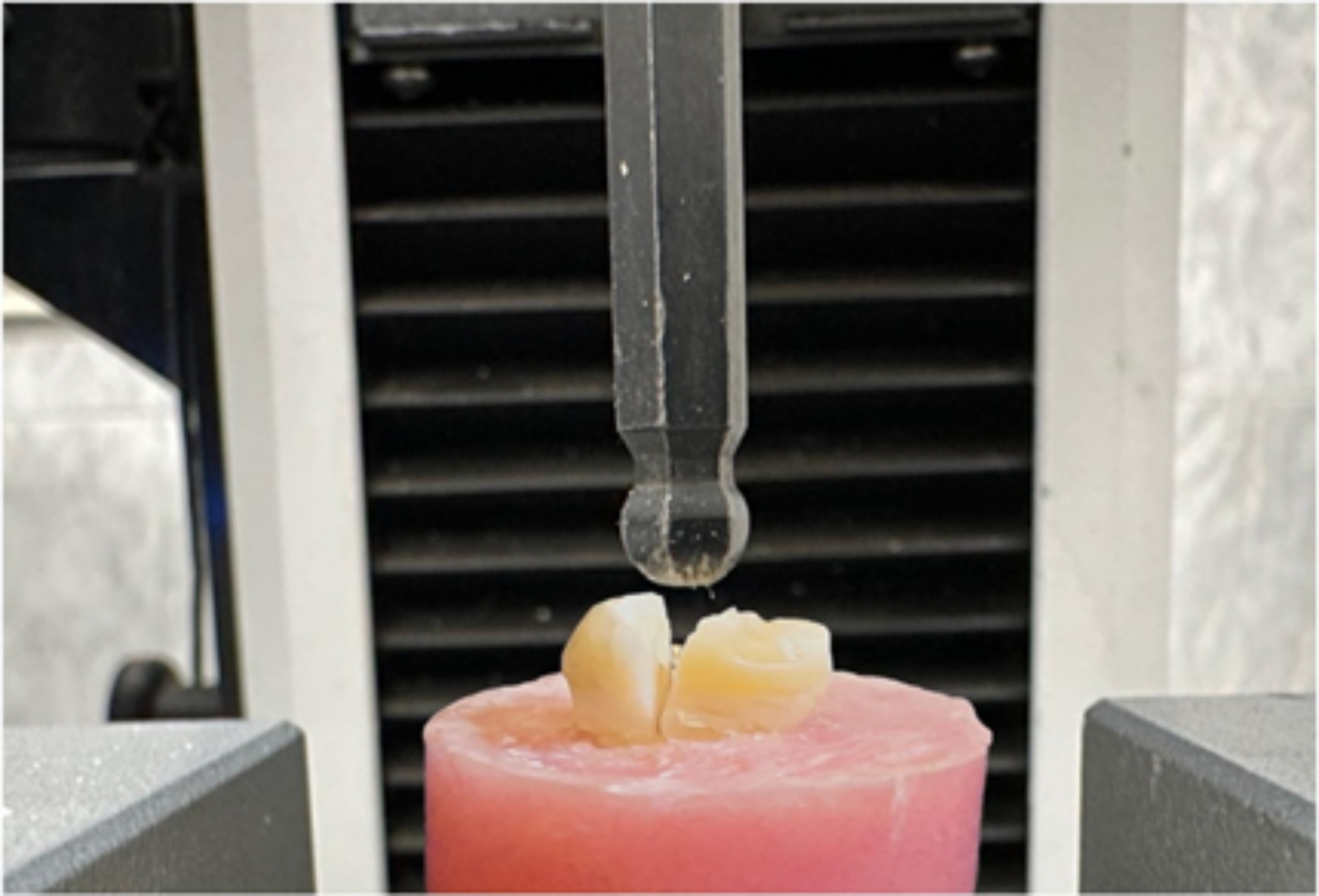
The load applied to the occlusal surface of the tooth until fracture

Each sample was fixed on a testing machine (Instron, Loyd Inst. Canton, MA, USA) that connected to a computer. All samples were screwed to the lower stabilized compartment of the machine while a ball-tipped metal cylinder was attached to the upper compartment of the machine. The ball tip of the cylinder, which was 4 mm in diameter, was held parallel to the long axis of the teeth, touching the samples occlusally at three points. Once the machine was turned on, a compressive load was applied parallel to the long axis of the teeth with a crosshead speed of 0.5 mm/min until the teeth were fractured as shown in Fig. 3. The computer software recorded the maximum force the teeth withstand and calculated it in Newton [25].

### Failure mode assessment

Two operators assessed the failure mode of the samples using a stereomicroscope and sorted them as:

### Type I failure

#### Restorable failure

**Fig. 4:**
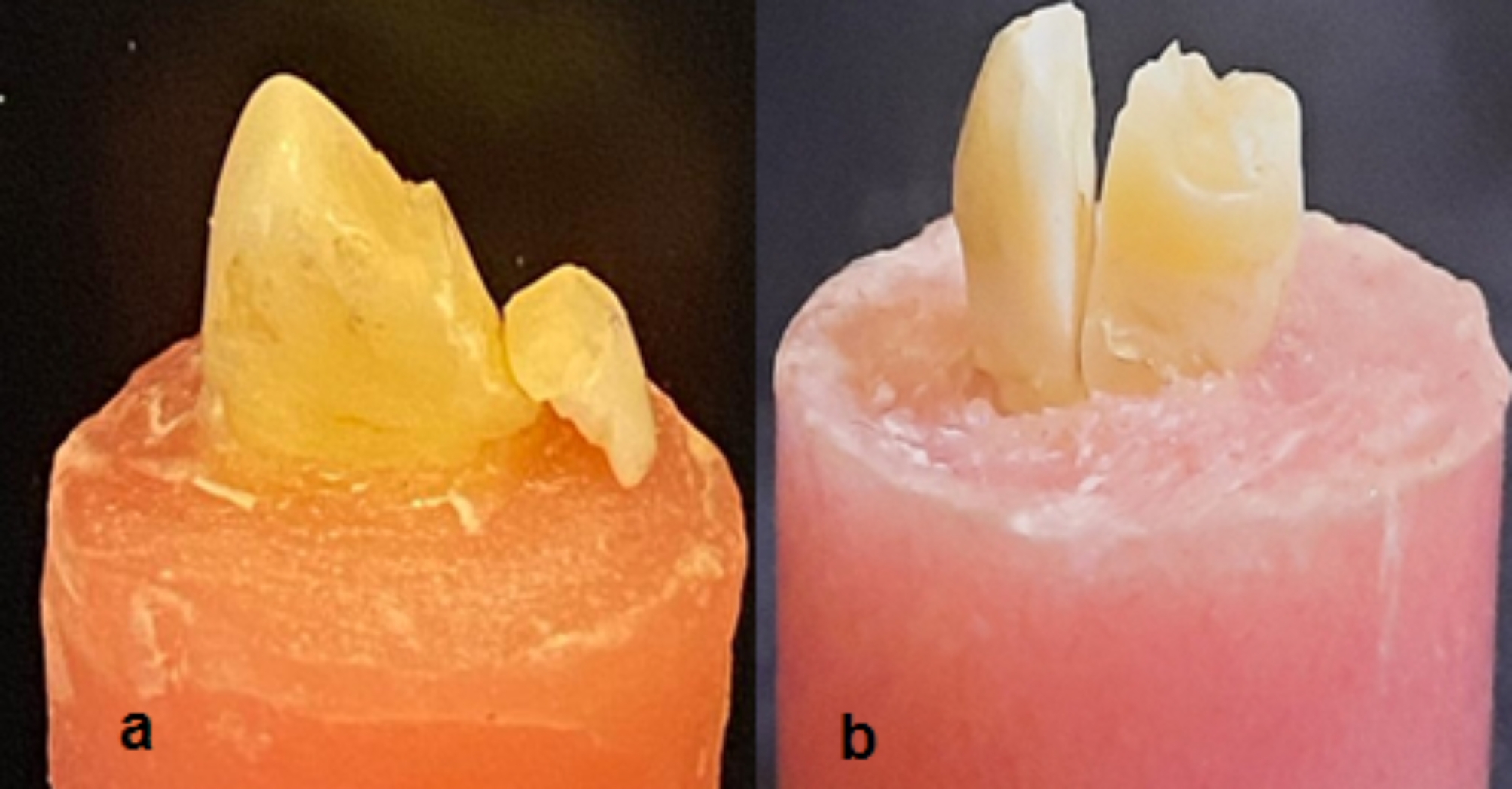
**(a)** Restorable failure, **(b)** Non-restorable failure

Fracture does not involve the root (Fig. 4a).

#### Non- restorable failure

Root is involved in fracture, and the fracture extends below the CEJ by more than 1 mm (Fig. 4b).

### Statistical analysis

Obtained data was analyzed using SPSS software, version 25 (SPSS Inc., PASW statistics for Windows version 25). The normality test was performed using the Shapiro-Wilk test. The mean and standard deviation of normally distributed fracture resistance data were calculated with a set significance of 0.05. A one-way ANOVA test was used to compare the obtained mean fracture load of all different independent groups. Then a post-hoc Tukey test was utilized to detect pair-wise comparison and determine significant differences among the groups. Qualitative data (mode of fracture) were described using numbers and percents. The Monte Carlo test was used to compare mode of fracture among all tested groups.

## Results

**Fig. 5:**
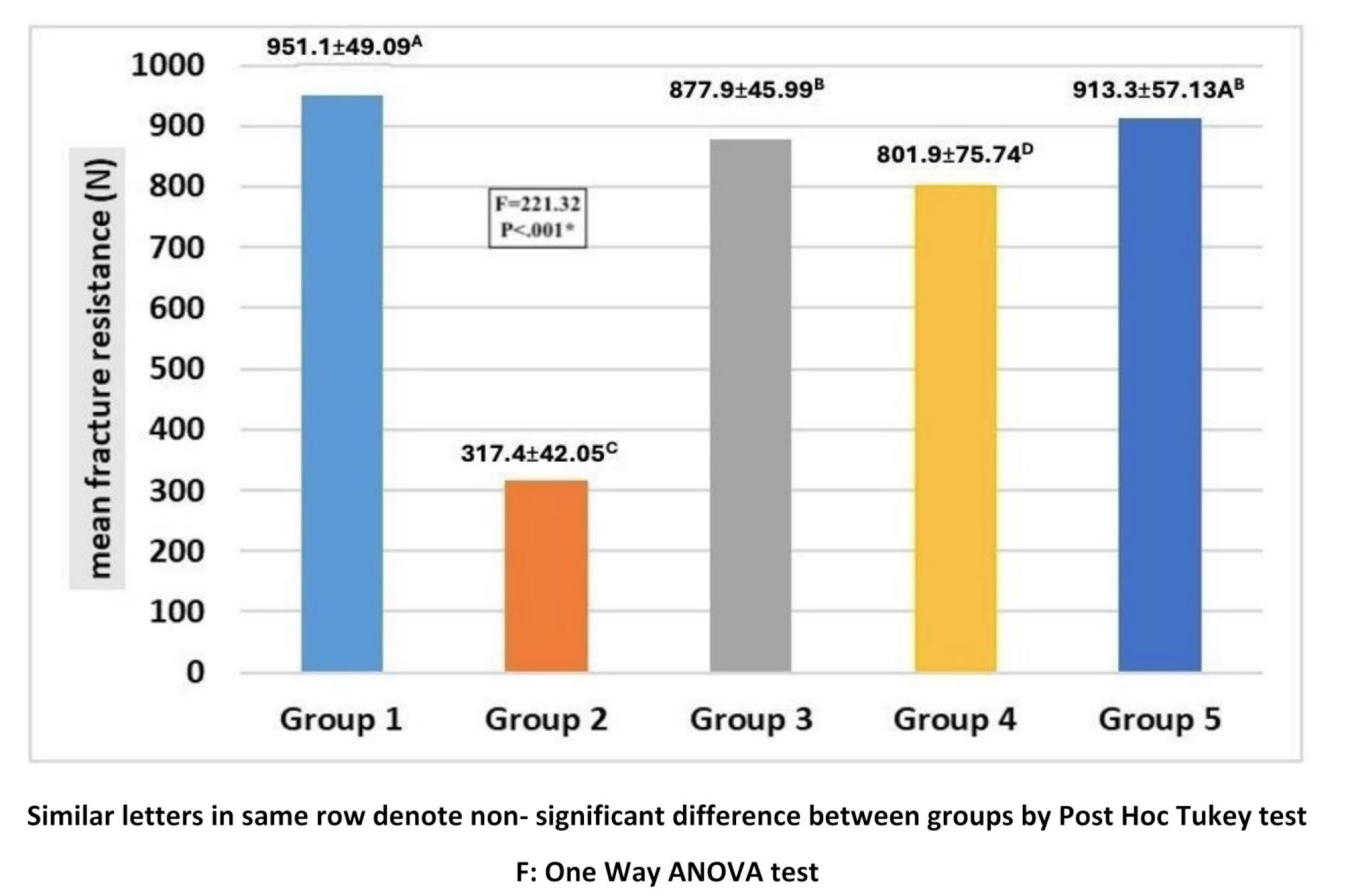
Diagram showing comparison of mean fracture resistance (*N*) between studied groups

The mean fracture loads of the different groups are presented in Fig. 5. One-way ANOVA (Table 2) showed that teeth restored with indirect overlay restorations recorded the highest fracture load (913 ± 57 N) among all restored teeth. No statistically significant differences (*p* > 0.05) were found in the mean fracture loads between intact teeth (positive control) (951.1 49.09 N) and teeth restored with indirect overlays (913 ± 57 N). Regarding teeth in group 3 that were restored with EverX Posterior, there was no significant difference between their mean fracture load (877.8 ± 45.99 N) and that of the teeth restored with indirect overlays. The teeth restored with EverX Posterior had significantly higher fracture resistance than those restored with EverX Flow (801.90 75.47 N).

The mode of failure for each of the tested groups is listed in Table 2. All groups showed two types of failures. Group 1 (intact teeth) showed the highest percentage of restorable fracture (80%), followed by group 5 restored with indirect overlays and group 4 restored with EverX Flow, which recorded the same percentage of restorable fracture (70%) with no significant difference between the three groups. On the other hand, the group restored with EverX Flow recorded a significantly higher percentage of restorable fractures (70%) than that restored with EverX Posterior (40%). The negative control group recorded the lowest percentage of restorable fractures (10%).

**Table 2:** Comparison of fracture mode (*N*) between studied groups

	Group 1	Group 2	Group 3	Group 4	Group 5	Test of significance
Restorable	8(80%)^ABC^	1(10%)^D^	4(40%)^ADEF^	7(70%)^BEG^	7(70%)^CFG^	χ^2MC^=13.37 *p* = 0.01*
Non-restorable	2(20%)	9(90%)	6(60%)	3(30%)	3(30%)	

Similar letters in same row denote non- significant difference between groups

MC: Monte Carlo test

## Discussion

Endodontic treatment of the teeth decreases its stiffness by only 5%, but MOD cavities decrease it by 69% [21]. This is mainly because of the absence of supporting walls and marginal ridges, followed by the subsequent increase in cuspal deflection and fracture [22]. Thus, in the current study, MOD cavity preparation was chosen to mimic the most catastrophic situation faced by teeth.

The upper bicuspid is the most potentially fracture-prone among posterior teeth. Due to their location in the arch, these teeth are subjected to compressive and shear loads. Considering their improper crown size, crown-root ratio, and anatomy, they have a tendency towards cuspal separation, and their resistance to fracture is challenging. Therefore, these teeth were selected for the current study since they provide an appropriate model for evaluating their fracture resistance when restored with the tested materials [21]. Considering that the teeth’s supporting structures significantly influence stress distribution [23], this study simulated the periodontal ligament and supporting bone.

To assess the fracture strength of different reinforced restorative resin composites, thermocycling followed by fracture resistance tests were performed. Thermal aging is conventionally used to simulate the in vivo aging of restorative materials by subjecting them to repeated cyclic exposures to hot and cold temperatures in water baths to reproduce thermal changes occurring in the oral cavity. Then, a static load is applied using an Instron testing machine. In the current study, the use of a metallic ball contacting the teeth in three points while applying load is claimed to create uniform contact with all cusps, whether functional or nonfunctional [24].

The applied load was directed along the teeth’s long axis, consistent with the approach used in previous studies to evaluate the fracture strength of different posterior teeth [23, 25].

This study used a conventional nanohybrid composite to overlay both EverX Posterior and EverX Flow, as advised by many authors. This approach aimed to implement the biomimetic technique concepts to get more uniform stress distribution and minimize polymerization shrinkage, which is greatly beneficial, especially in extensive restorations and areas of high stress [26, 27].

For enhancement of the strength of teeth subjected to endodontic therapy, cuspal coverage is usually recommended [28]. As concluded by the authors, overlays that cover all cusps may provide a favorable stress distribution and enhance the fracture resistance of teeth [29, 30]. Accordingly, overlay design preparation with all cuspal coverage was selected to be tested in this study.

The null hypothesis of this study was rejected because of the significant differences in fracture resistance and failure mode between the two fiber reinforced composite groups. The mean fracture strength value of intact premolars was 951.1 ± 49.09 N, which is acceptable and within the range obtained in previous studies (882–1568 N) [30, 31]. These intact teeth showed the maximum fracture strength value compared to the other groups. This could be explained by the preservation of healthy tooth structure. Regarding the insignificant difference in fracture strength of these intact teeth and the teeth restored with indirect ceramic cuspal coverage, as obtained by the current study, this may be explained by the good compressive strength of ceramic materials that are valuable restorations in stress-bearing areas [27]. This is in agreement with Cubas et al. [28], Stappert et al. [29], and Yoon et al. [30], who concluded that the fracture resistance of intact teeth was unaffected by cuspal coverage. While Elassar et al. [31] and Harsha et al. [32] demonstrated that cuspal coverage had a substantial positive effect on the fracture resistance of teeth when compared to sound teeth, these results differ from the current study findings.

On the other hand, the insignificant difference in mean fracture load between the teeth restored with short fiber-reinforced composite (EverX Posterior) and those restored with CeraSmart overlays in the current study may be attributable to the randomly oriented short E-glass fiber fillers included in EverX Posterior, which could positively affect the fracture resistance [33]. This effect is due to their “critical fiber length,” defined as the minimum length where the material’s shear strength reaches the maximum value when the fiber center reaches its optimum tensile strength. As provided by the literature, 0.5–1.6 mm should be the minimum length of the fiber to exhibit enhanced properties. The average length of E-glass fibers is 1–2 mm; this could explain why EverX Posterior, in the current study, showed comparable fracture strength to the indirect ceramic overlays. These fibers can also act as crack propagation stoppers and distribute the stresses uniformly, as concluded by some literature [34, 35]. Garoushi et al. concluded that the mechanical properties and load-withstanding ability of resin composite are significantly enhanced when randomly oriented multidirectional short glass fibers are incorporated in it, as they behave as a stopper of crack propagation [36].

Some factors significantly affect the discontinuous fiber-reinforced composite’s mechanical characteristics. The most significant parameters that could enhance or decrease the mechanical characteristics of a discontinuous fiber-reinforced composite are aspect ratio, critical fiber length, fiber loading, and fiber orientation [37]. EverX Flow, as a new low aspect ratio fiber reinforced material, was not able to match the performance of its high aspect ratio version, EverX Posterior. This can be attributed to the difference in fiber length and aspect ratio between the two materials. Furthermore, the barium glass filler content in EverX Flow was reduced to create a flowable form. This decrease in the filler content is responsible for reducing the modulus of elasticity of this flowable material, which in turn may adversely affect the fracture toughness [37]. Moreover, modifying the resin matrix of EverX Flow may account for the insignificant difference in fracture resistance of the two short fiber-reinforced composites, as the polymeric matrix structure may influence the mechanical characteristics of dental composites. Besides, the stress transfer from the matrix to the filler plays a crucial role in determining the material’s mechanical properties. On the other hand, the bonding between the resin and filler may also differ as the matrix differs [13].

The results of the two short fiber-reinforced composite groups align with the findings of Mange et al. [37], who demonstrated that EverX Posterior-restored teeth showed greater fracture resistance than EverX Flow-restored teeth. However, these findings contrast with the data provided by Lassilla et al., who concluded that teeth restored with EverX Flow had higher fracture resistance than those restored with EverX Posterior [18].

Regarding the fracture mode, the group restored with EverX Flow reported an 80% restorable fracture rate, comparable to the intact teeth group that recorded a 90% rate of restorable fracture. This may be attributed to the enhanced flow, lower modulus of elasticity, and fewer cuspal strains of flowable composites, as documented by several authors [38, 39]. The non-significant difference between the two groups may result from the addition of short fibers, which increases the viscosity of the material and favors the failure of the adhesive interface [39].

The EverX Posterior group exhibited a non-restorable fracture (70%), significantly higher than the EverX Flow group. This may be explained by the former’s increased viscosity and elasticity modulus. These results align with Magne et al., who demonstrated more restorable fracture with EverX Flow and more non-restorable fracture with EverX Posterior [37]. The short fiber-reinforced material’s resistance to fracture demonstrates a distinctive variety of fiber and polymer, which results in a wide range of improved mechanical and physical characteristics.

The biomimetic restorative approach is a recommended direct restoration option that can be reliably utilized for crown reconstructions of teeth with substantial structure loss and subjected to high stress. This technique employs a short fiber-reinforced composite as a core material covered with a conventional composite. To achieve optimal durability and reinforce advantages, it is essential to adhere to the correct application techniques for this material. One of the limitations of the present study is that a static impactive fracture load was applied and determined; in contrast, stresses applied to teeth and restorations are typically cyclic in nature and lower in value. Nevertheless, this static testing approach provides valuable insights into fracture mode and strength, highlighting the relative relationship between static and cyclic loading [40]. Another limitation of this study is the absence of a conventional composite group and the small sample size, which should be considered in further studies. Clinical studies are still recommended to assess the durability of these restorations.

## Conclusions

Within the limitations of this study, it was concluded that:

1. The packable short-fiber reinforced composite EverX Posterior has an adequate degree of fracture resistance, indicating that it would be an appropriate material for the restoration of endodontically treated teeth that have extensive cavities.
2. The flowable fiber reinforced composite EverX Flow has lower fracture resistance than high aspect ratio one, but it exhibited more favorable fractures.

## Data Availability

Available upon request from the corresponding author Dr. Hafez M.
